# Stand-alone versus supplemented ALIF: a systematic review and meta-analysis of pseudarthrosis and reoperation rates

**DOI:** 10.1007/s10143-026-04347-1

**Published:** 2026-06-03

**Authors:** Ricardo Salemi Riechelmann, Franz Jooji Onishi, Telmo Augusto Barba Belsuzarri, Sérgio Cavalheiro, Eduardo Augusto Iunes

**Affiliations:** 1 Associação de Assistência à Criança Deficiente (AACD), São Paulo, São Paulo Brazil; 2 Department of Neurosurgery, Federal University of São Paulo (UNIFESP), Rua Borges Lagoa 1080, Sala 1405, São Paulo, São Paulo Brazil; 3 Department of Neurosurgery, Pontifical Catholic University of Campinas (PUC- Campinas), Campinas, São Paulo Brazil

**Keywords:** Anterior lumbar interbody fusion, Pseudarthrosis, Lumbar vertebrae, Spinal fusion, Pedicle screws, Degenerative disc disease

## Abstract

Anterior lumbar interbody fusion (ALIF) is widely used for degenerative lumbar disc disease, offering restoration of disc height and sagittal alignment. However, pseudarthrosis remains a relevant complication, and the benefit of supplemental posterior fixation over stand-alone constructs is still debated. To compare the incidence of pseudarthrosis following stand-alone ALIF versus ALIF combined with posterior pedicle screw fixation. A systematic review and meta-analysis were conducted in accordance with PRISMA guidelines and registered in PROSPERO (CRD42024581358). PubMed/MEDLINE, Embase, Cochrane Library, and BVS were searched for studies published between 2013 and September 2024. Clinical studies including patients with degenerative lumbar disease undergoing ALIF with at least 12 months of follow-up were eligible. Study selection and data extraction were performed independently by two reviewers. Risk of bias was assessed using the Joanna Briggs Institute (JBI) tool for observational studies and Risk of Bias 2 (RoB 2) for randomized trials. Random-effects models were used to estimate pooled pseudarthrosis prevalence and clinical outcomes. Fourteen studies comprising 917 patients and over 1,000 operated levels were included. The pooled prevalence of pseudarthrosis was 8.17% (95% CI: 5.04–11.31), with substantial heterogeneity (I²=70.8%). Pseudarthrosis rates were numerically higher in stand-alone ALIF (8.95%; 95% CI: 4.54–13.37) compared with ALIF combined with posterior fixation (6.76%; 95% CI: 3.58–9.94), although the difference was not statistically significant (*p* = 0.429). Significant clinical improvement was observed, with a mean reduction of 4.16 points in VAS and a 24.46-point improvement in ODI. Meta-regression demonstrated no association between pseudarthrosis rates and clinical outcomes. Notably, stand-alone ALIF was associated with a significantly higher risk of reoperation for symptomatic pseudarthrosis (RR 6.8; 95% CI: 1.9–24.5; *p* < 0.01). ALIF provides substantial pain relief and functional improvement, with a relatively low overall rate of pseudarthrosis. Although stand-alone constructs showed a trend toward higher pseudarthrosis rates without statistical significance, they were associated with a markedly increased risk of reoperation. These findings suggest that, while fusion rates may appear comparable, supplemental posterior fixation may confer greater mechanical reliability and reduce clinically meaningful failure in selected patients.

**Supplementary Information:**

The online version contains supplementary material available at 10.1007/s10143-026-04347-1.

## Introduction

Lumbar spinal fusion is a well-established surgical strategy for the treatment of degenerative lumbar spine disorders. Despite advances in surgical techniques and implant technology, pseudarthrosis—defined as the absence of solid bone fusion—remains a clinically relevant complication associated with persistent pain, functional impairment, and the need for revision surgery [1–3]. Its development is multifactorial, involving construct stability, implant characteristics, graft material, and patient-related factors such as smoking and bone quality [4–8].

Computed tomography (CT) is considered the most reliable modality for diagnosing pseudarthrosis, although dynamic radiographs are commonly used in clinical practice [1, 6]. However, radiographic findings do not consistently correlate with clinical symptoms, and treatment decisions are primarily guided by clinical evaluation [3].

To improve fusion rates and biomechanical stability, interbody fusion techniques were developed, including posterior lumbar interbody fusion (PLIF) and transforaminal lumbar interbody fusion (TLIF), as well as more recent lateral approaches such as LLIF and OLIF [2, 4, 9]. Anterior lumbar interbody fusion (ALIF), originally described in the early 20th century [10], has gained renewed interest due to its ability to restore disc height and lumbar lordosis while preserving posterior elements. However, the anterior approach is associated with specific risks, particularly vascular injury, which can be minimized with appropriate surgical expertise [2, 4, 10].

The addition of posterior pedicle screw fixation has historically improved construct stability and fusion rates. However, with advances in interbody cage design, the necessity of routine supplemental posterior fixation after ALIF remains controversial. Stand-alone ALIF may provide sufficient stability in selected patients, whereas combined anterior-posterior constructs may enhance fusion rates but at the cost of increased surgical morbidity, operative time, and healthcare costs.

Therefore, the present study aims to compare the incidence of pseudarthrosis following stand-alone ALIF versus ALIF with supplemental posterior fixation in patients with degenerative lumbar spine disease, to determine whether increased construct stability translates into improved fusion outcomes.

## Objectives

### Study hypothesis

The primary hypothesis is that stand-alone ALIF may have a higher incidence of pseudarthrosis than ALIF supplemented with posterior pedicle screw fixation, reflecting the increased biomechanical stability provided by circumferential constructs.

### Primary objective

To compare the incidence of pseudarthrosis in patients with degenerative lumbar spine disease treated with anterior lumbar interbody fusion (ALIF) performed as a stand-alone procedure versus ALIF supplemented with posterior pedicle screw fixation.

### Secondary objectives

1. To evaluate the clinical outcomes associated with ALIF with and without supplemental posterior fixation, including improvement in pain, functional status, and quality of life as measured by the Visual Analog Scale (VAS) and Oswestry Disability Index (ODI).
2. To describe and compare postoperative complication rates between the two surgical strategies, categorizing complications as access-related, implant-related, and systemic complications.

The research question was structured according to the according to the Population, Intervention, Comparison, Outcome (PICO) framework: adult patients with degenerative lumbar spine disease (P) undergoing ALIF with supplemental posterior fixation (I) compared with stand-alone ALIF (C), evaluating pseudarthrosis incidence and clinical and radiological outcomes (O).

## Materials and methods

### Search strategy

This systematic review and meta-analysis were conducted in accordance with Preferred Reporting Items for Systematic Reviews and Meta-Analyses (PRISMA) guidelines with protocol registered in the International Prospective Register of Systematic Reviews (PROSPERO) (CRD42024581358). A comprehensive literature search was performed on September 24, 2024, in the following databases: PubMed/MEDLINE, Embase, Cochrane Library, and BVS.

Studies published between January 2013 and September 2024 were considered, reflecting the period of increasing clinical adoption of anterior lumbar interbody fusion (ALIF). The search strategy used combinations of controlled vocabulary and keywords related to “anterior lumbar interbody fusion” (ALIF) and “pseudarthrosis”, constructed using DeCS/MeSH descriptors. The full search strategy is provided in Appendix 1.

**Fig. 1:**
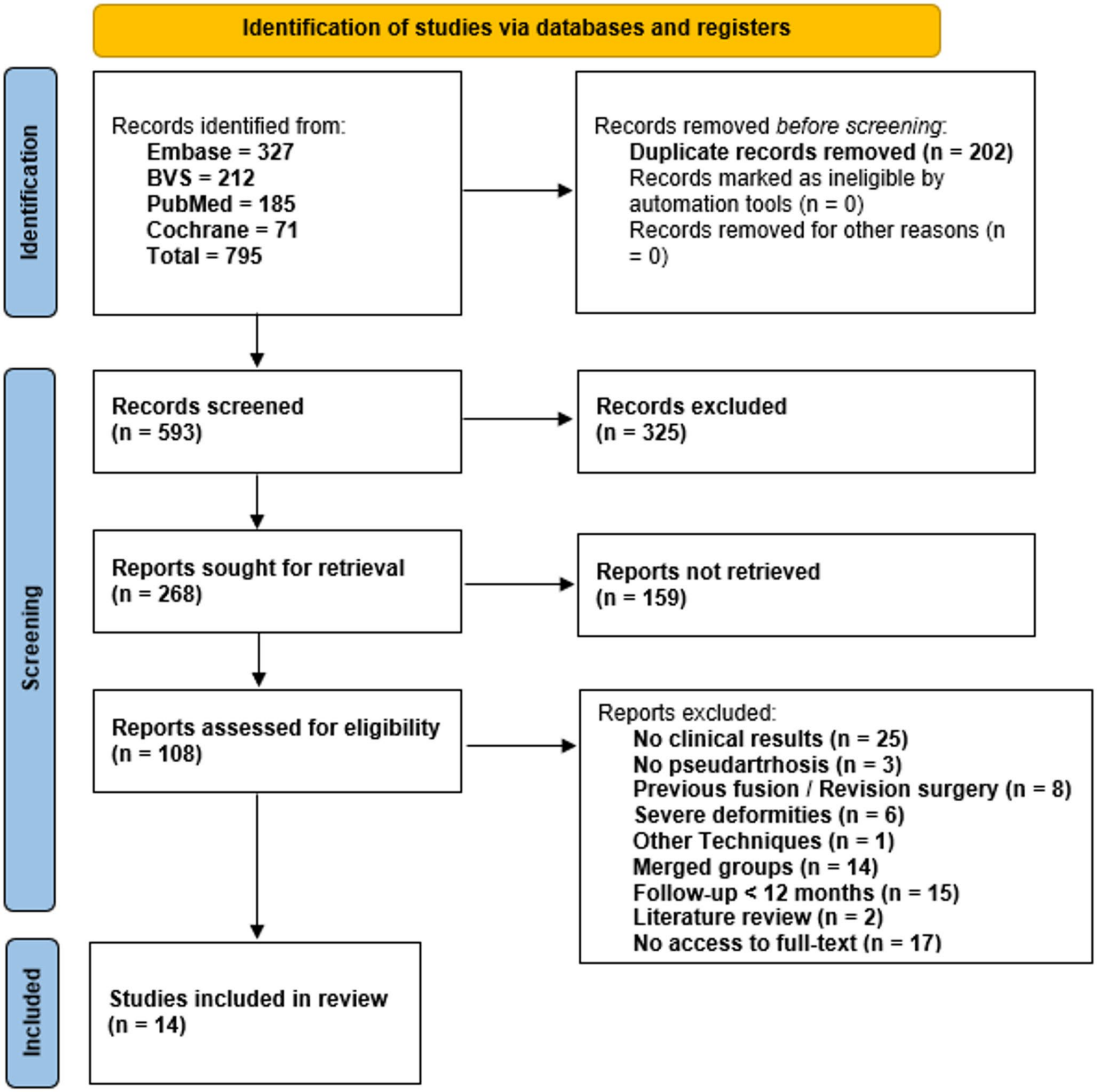
Prisma flow diagram

Study selection was performed independently by two reviewers, who screened titles and abstracts for relevance. Full-text articles were subsequently assessed for eligibility. Disagreements were resolved by a third independent reviewer. The study selection process is summarized in a PRISMA flow diagram (Fig. 1).

### Eligibility criteria

Studies were included if they:

- involved adult patients with degenerative lumbar spine disease undergoing ALIF,
- reported clinical outcomes and pseudarthrosis incidence,
- had a minimum follow-up of 12 months, and
- were clinical, observational, or descriptive studies published between 2013 and 2024.

Studies evaluating stand-alone ALIF or ALIF combined with posterior pedicle screw fixation were eligible.

Studies were excluded if they:

- involved a previous lumbar fusion,
- included other interbody fusion techniques combined with ALIF,
- used posterior fixation methods other than pedicle screws, or
- primarily involved patients with degenerative lumbar scoliosis greater than 30°.

### Data extraction

Data extraction was independently performed by two reviewers using the Rayyan platform (rayyan.ai), with discrepancies resolved by a third reviewer. Extracted variables included patient characteristics, underlying pathology, surgical technique, graft material, fixation strategy, and clinical outcomes. The primary outcome was pseudarthrosis rate, comparing stand-alone ALIF with ALIF supplemented by posterior pedicle screw fixation. Secondary outcomes included postoperative complications and clinical outcomes.

### Risk of bias assessment

Methodological quality of the included observational studies was assessed independently by two reviewers using the Joanna Briggs Institute (JBI) Critical Appraisal Tool. For randomized controlled trials, risk of bias was evaluated using the Cochrane Risk of Bias 2 (RoB 2) tool. Detailed results of the risk of bias assessment are provided in Appendix 2.

### Statistical analysis

Descriptive statistics were used to summarize study characteristics. Pooled prevalence of pseudarthrosis was calculated using random-effects meta-analysis models with the inverse variance method, considering the expected heterogeneity among studies.

Subgroup analyses were conducted according to:

- fixation strategy (stand-alone vs. supplemental posterior fixation),
- type of graft material, and
- number of fused levels.

For continuous clinical outcomes, including Visual Analog Scale (VAS) and Oswestry Disability Index (ODI), pooled mean differences between preoperative and postoperative values were calculated.

Statistical heterogeneity was assessed using the I² statistic and visually inspected through forest plots. Meta-regression analysis was additionally performed to investigate whether the proportion of pseudarthrosis influenced postoperative improvement in VAS and ODI scores.

All analyses were conducted using RStudio, with the metafor package used for meta-analyses and graphical outputs.

## Results

Fourteen studies published between 2014 and 2023 were included (Fig. 1), representing multiple geographic regions [11–24]. Most studies originated from Asia (6 studies; 42.9%), followed by Europe (4 studies; 28.6%), the Americas (2 studies; 14.3%), and Oceania (1 study; 7.1%). The most frequently represented countries were South Korea (3 studies; 21.4%), France (3 studies; 21.4%), and China (2 studies; 14.3%), while Japan, the Netherlands, Australia, and the United States each contributed one study (7.1%).

Regarding study design, retrospective cohort studies were the most common (6 studies; 42.9%), followed by retrospective case series (4 studies; 28.6%), prospective case series (2 studies; 14.3%), and randomized controlled trials (2 studies; 14.3%). The main characteristics of the included studies are summarized in Table 1.

**Table 1:** Characteristics of Included Studies

Study	Continent	Country	Total Patients (*n*)	Mean Age (years)	Predominant Sex	Follow-up (months)	ALIF Procedures (*n*)	Fusion (*n*)	Fusion Rate (%)	Pseudarthrosis (*n*)	Pseudarthrosis (%)
Kimura et al., 2014 [11]	Asia	Japan	42	36	Male	24	42¹	35	83.3	7	16.7
Lavelle et al., 2014 [12]	–	–	73	44.2	Female	24	73¹	30	41.1	10	13.7
Allain et al., 2014 [13]	Europe	France	65	57.1	Female	12	65¹	54	83.1	2	3.1
Lee et al., 2020 [14]	Asia	South Korea	53	72.1	Female	64	53²	51	96.2	2	3.8
Schimmel et al., 2016 [15]	Europe	Netherlands	95	43.4	Female	47.7	95¹	72	75.8	23	24.2
Lee et al., 2017 [16]	Asia	South Korea	26	53.2	Female	24	26¹	23	95.8	3	12.5
Kuang et al., 2017 [17]	Asia	China	42	52.9	Female	24.5	42¹	42	100	0	0
Kleimeyer et al., 2018 [18]	America	USA	42	50.1	Male	82.8	62¹	38	61.3	4	6.5
Mobbs et al., 2018 [19]	Oceania	Australia	30	46	Male	240	35¹	31	88.6	3	8.6
Gornet et al., 2019 [20]	America	USA	119	40.2	–	60	172¹	161	93.6	11	6.4
Szadkowski et al., 2021 [21]	Europe	France	48	46.9	Female	18.2	48¹	41	85.4	4	8.3
Chung et al., 2021 [22]	Asia	South Korea	45	61.9	Female	42.8	45²	42	100	3	7.1
Tung et al., 2023 [23]	Asia	China	69	55.3	Female	24	84²	78	92.9	6	7.1
Ould-Slimane et al., 2023 [24]	Europe	France	115	46.9	Male	46	75²	66	88.0	9	12.0

¹ Stand-alone anterior lumbar interbody fusion (ALIF)

² ALIF combined with posterior pedicle screw fixation (circumferential fusion)

Across the 14 studies, a total of 864 patients underwent ALIF procedures, representing at least 1,059 operated levels. One study [24] did not specify whether the procedures involved single-level or multilevel fusion. Study sample sizes ranged from 26 to 119 patients, with a mean age of 52.7 years. On average, each study included 62 patients (SD ± 30) and 73 operated levels (SD ± 41)(Table 1).

**Fig. 2:**
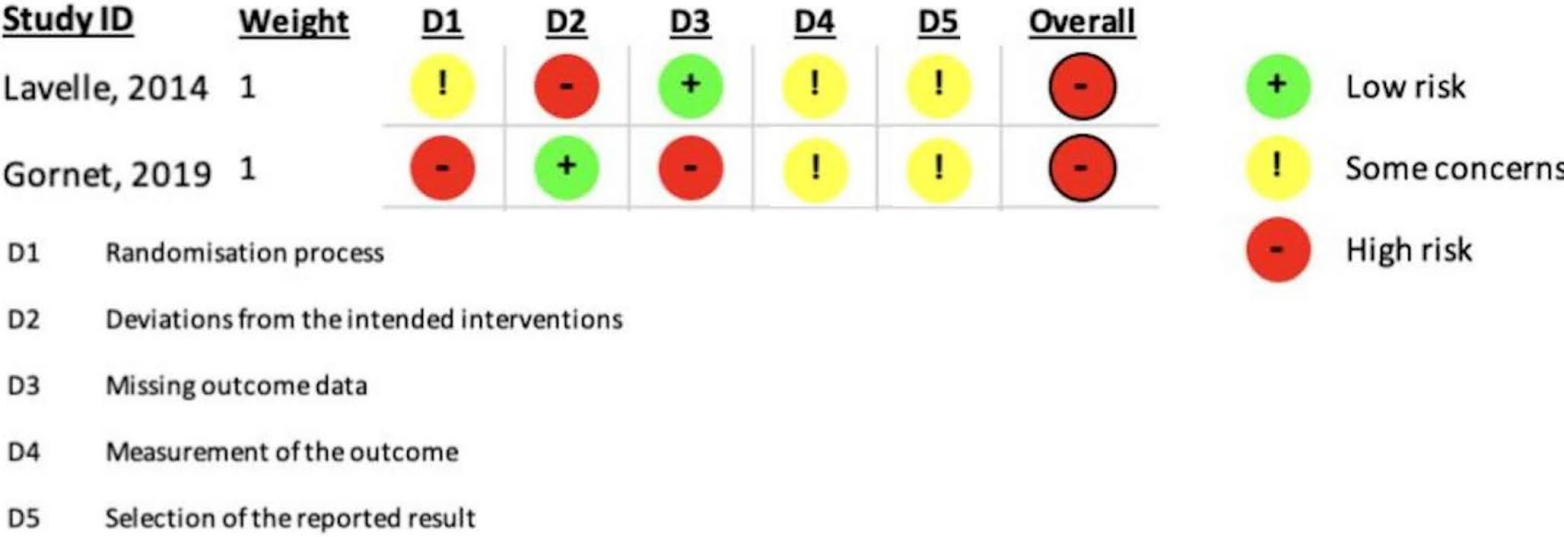
ROB2 assessment of the RCTs

### Risk of bias assessment

The risk of bias for randomized controlled trials was assessed using the RoB 2 tool (Fig. 2). Both included RCTs were judged to have a high overall risk of bias, primarily due to missing outcome data, limited methodological reporting, and concerns regarding selective reporting.

In addition, the methodological quality of the included observational studies was assessed using the Joanna Briggs Institute (JBI) Critical Appraisal Tool (Table 2). Overall, studies demonstrated moderate to high quality, with most domains adequately addressed. The main methodological limitations concerned the identification and control of confounding factors, as well as variability in outcome measurement, suggesting potential residual bias and heterogeneity across studies.

**Table 2.**
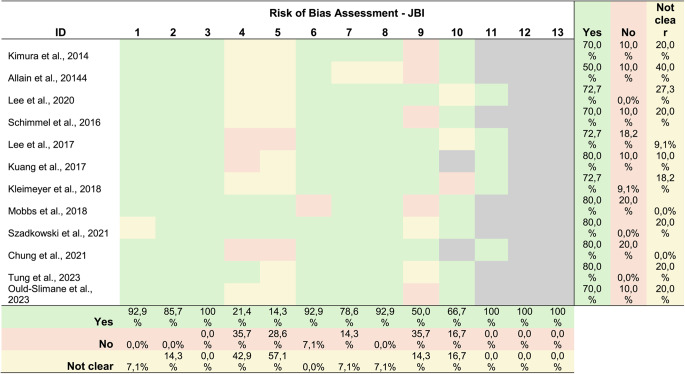
Risk of bias assessment of included studies using the Joanna Briggs Institute (JBI) Critical Appraisal Tool for observational studies

The included studies predominantly involved patients presenting with low back pain, while intermittent claudication was rarely reported. Degenerative disc disease was the most common underlying diagnosis, followed by spondylolisthesis, whereas lumbar stenosis was less frequently represented. The mean duration of symptoms was approximately 9 months, reflecting a chronic disease profile.

A total of 917 ALIF procedures were analyzed, most of which were performed as stand-alone constructs, with the majority involving single-level fusion. The most frequently treated segments were L5–S1 and L4–L5, accounting for over 90% of cases. Overall fusion was achieved in the majority of patients, with pseudarthrosis reported in approximately 8% of cases.

PEEK cages and autologous grafts were the most commonly used materials, while alternative grafts such as BMPs, bioceramics, and allografts were less frequently applied. Considerable variability was observed in fixation strategies and postoperative management, including the use of supplemental fixation, bracing, and surgical team composition.

These characteristics are summarized in Table 3.

**Table 3:** Descriptive statistics of surgical and procedural characteristics across the included studies

Variable / Category	Studies (*n*)	% (95% CI)
Surgical team		
Multiple teams	5	38.46 (13.86–68.42)
Single team	8	61.54 (31.58–86.14)
Cage material		
Other	1	8.33 (0.21–38.48)
PEEK	9	75.00 (42.81–94.51)
Titanium	2	16.67 (2.09–48.41)
Graft type		
Allograft	1	8.33 (0.21–38.48)
Autograft	9	75.00 (42.81–94.51)
Bioceramic	1	8.33 (0.21–38.48)
BMP	1	8.33 (0.21–38.48)
Cage fixation method		
Two screws	1	8.33 (0.21–38.48)
Four screws	2	16.67 (2.09–48.41)
Anchors	3	25.00 (5.49–57.19)
None	4	33.33 (9.92–65.11)
Single screw	2	16.67 (2.09–48.41)
Stand-alone		
No	14	100 (76.84–100)
Access surgeon involvement		
No	5	83.33 (35.88–99.58)
Yes	1	16.67 (0.42–64.12)
Postoperative brace use		
No	4	57.14 (18.41–90.10)
Yes	3	42.86 (9.90–81.59)

### Visual analog scale (VAS)

**Fig. 3:**
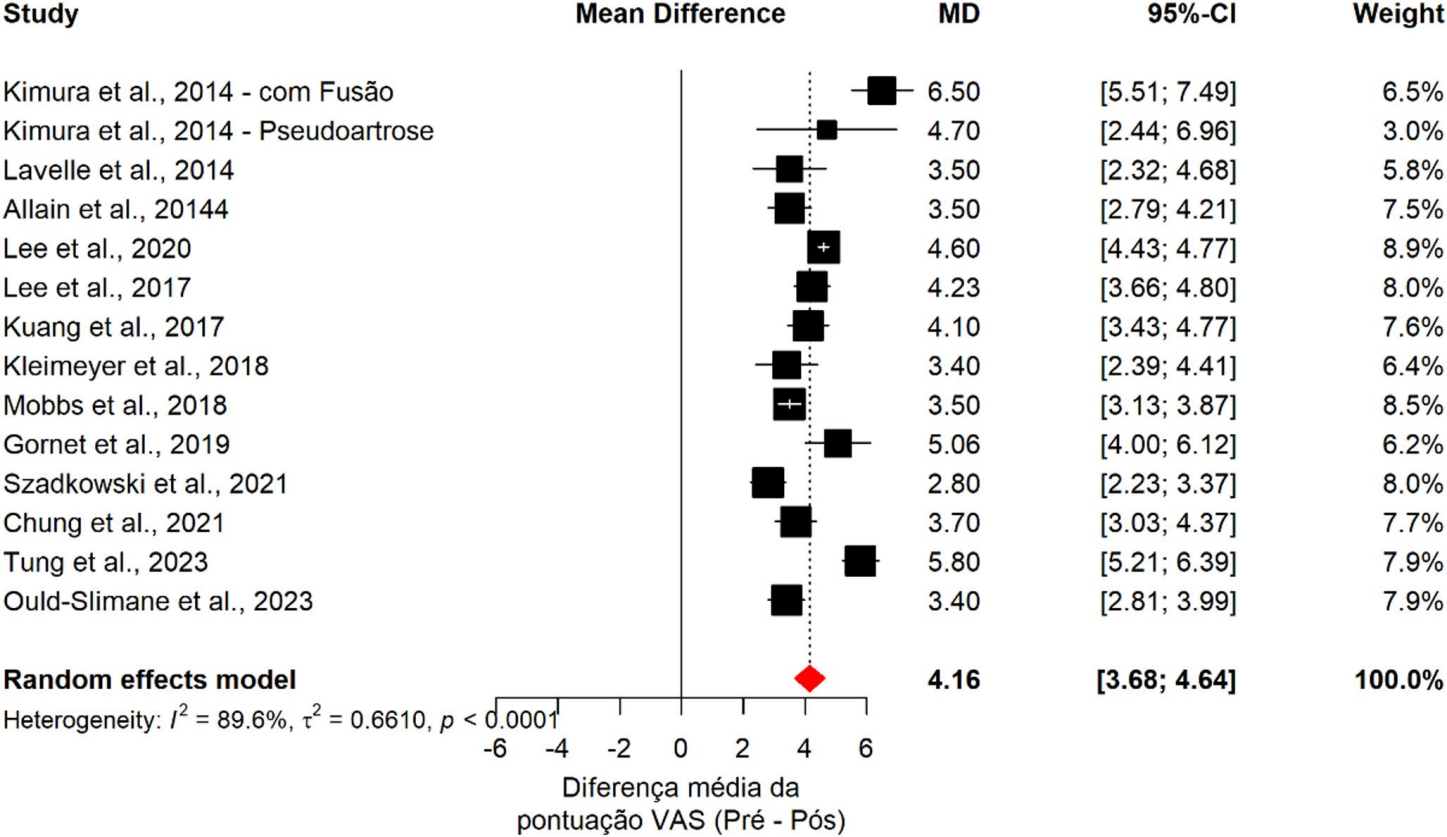
Forest plot of the meta-analysis showing the pooled mean difference in Visual Analog Scale (VAS) scores between preoperative and postoperative assessments

Although not a primary outcome of this study, pooled analysis of 14 studies demonstrated a significant reduction in pain following ALIF, with a mean improvement of 4.16 points in VAS scores. However, substantial heterogeneity was observed (I² = 89.6%), reflecting variability in patient populations, surgical techniques, and follow-up protocols across studies (Fig. 3).

Meta-regression analysis showed no significant association between pseudarthrosis rates and pain improvement (β = −0.0382; 95% CI: −2.91 to 2.83; *p* = 0.9792). Residual heterogeneity remained high (I² = 89.93%), with no variability explained by pseudarthrosis (R² = 0%), suggesting that nonunion was not associated with the magnitude of VAS improvement following ALIF.

Importantly, no statistically significant differences in pain improvement were identified according to fixation strategy (stand-alone vs. anterior-posterior ALIF), number of fused levels, or graft type, indicating consistent clinical benefit across different surgical approaches.

At the individual study level, pain reduction ranged from 2.8 to 6.5 points.

### Oswestry disability index (ODI)

**Fig. 4:**
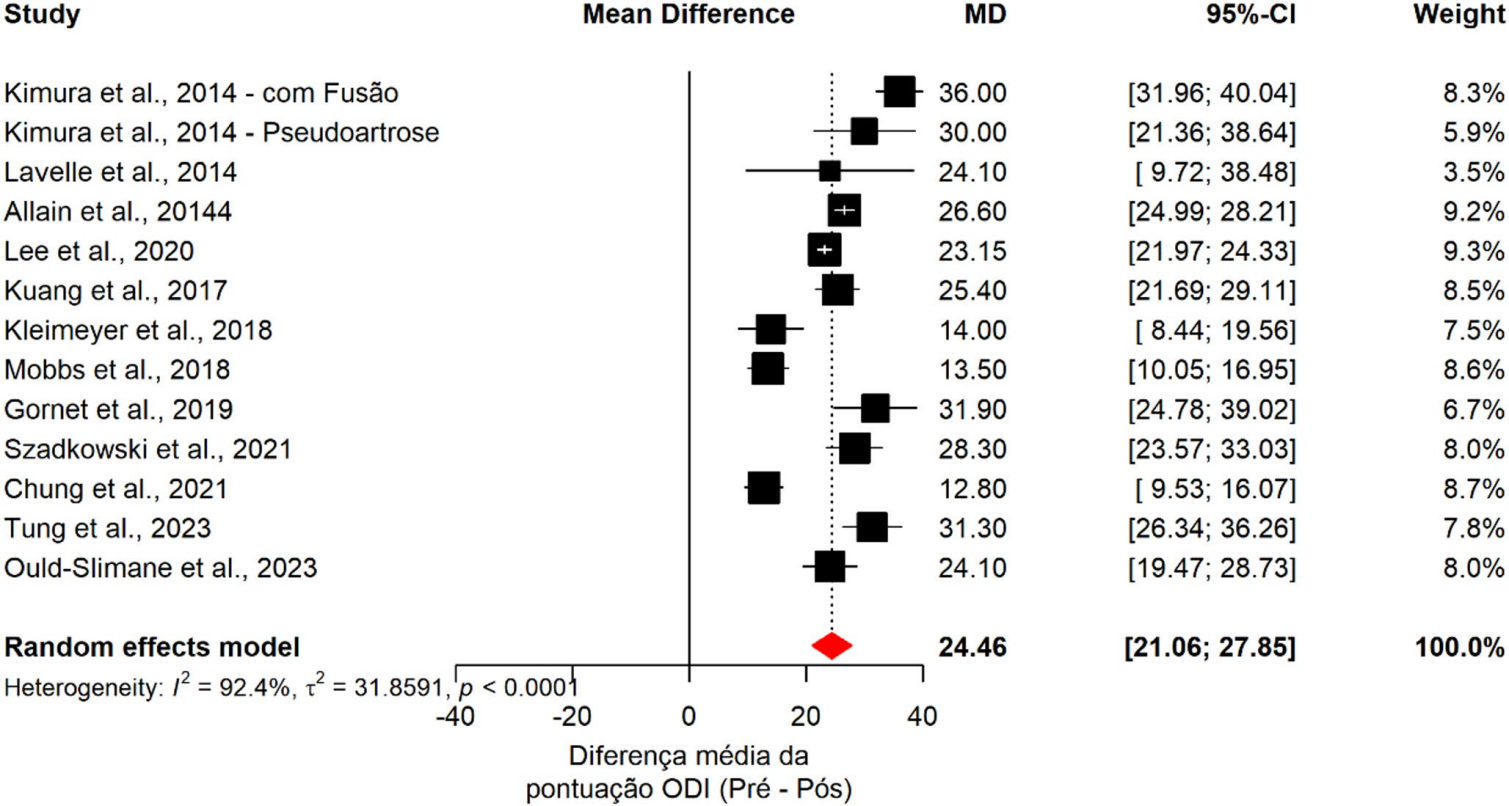
Forest plot showing the pooled mean difference in Oswestry Disability Index (ODI) scores between preoperative and postoperative time points following ALIF. Squares represent individual study estimates, and the diamond indicates the pooled effect with corresponding 95% confidence intervals

Pooled analysis of 13 studies (849 patients) demonstrated significant functional improvement following ALIF, with a mean ODI reduction of 24.46 points (95% CI: 21.06–27.85; *p* < 0.001). Substantial heterogeneity was observed (I² = 89.6%) (Fig. 4).

Meta-regression analysis showed no significant association between pseudarthrosis rates and functional improvement (ODI) (*p* = 0.586). Residual heterogeneity remained high (I² = 93.0%), with no variability explained by pseudarthrosis (R² = 0%), indicating that nonunion did not influence the magnitude of functional recovery.

No significant differences in ODI improvement were identified according to fixation strategy (stand-alone vs. anterior-posterior ALIF; *p* = 0.491), number of fused levels (*p* = 0.416), or graft type (*p* = 0.6412), indicating consistent functional benefit across different surgical approaches.

### Pseudoartrosis analysis

Pseudarthrosis was most commonly diagnosed using computed tomography (CT), followed by dynamic and plain radiographs, while MRI was infrequently used (Table 4). The most frequently reported management strategy was posterior spinal fusion with pedicle screw fixation, whereas conservative treatment was rarely described. Reoperation for pseudarthrosis was reported in most studies and was markedly more frequent in stand-alone ALIF compared to circumferential constructs.

**Table 4:** Descriptive statistics of diagnostic methods and management of pseudarthrosis across included studies

Variable / Category	Studies (*n*)	% (95% CI)
Plain radiography used for diagnosis		
No	2	28.57 (3.67–70.96)
Yes	5	71.43 (29.04–96.33)
Dynamic radiography used for diagnosis		
No	2	25.00 (3.19–65.09)
Yes	6	75.00 (34.91–96.81)
Computed tomography (CT) used for diagnosis		
No	2	20.00 (2.52–55.61)
Yes	8	80.00 (44.39–97.48)
Magnetic resonance imaging (MRI) used for diagnosis		
No	2	66.67 (9.43–99.16)
Yes	1	33.33 (0.84–90.57)
Management of pseudarthrosis		
Conservative treatment	1	12.50 (0.32–52.65)
Posterior fusion with pedicle screw fixation	6	75.00 (34.91–96.81)
Other	1	12.50 (0.32–52.65)
Reoperation reported		
No	2	25.00 (3.19–65.09)
Yes	6	75.00 (34.91–96.81)

Pseudarthrosis occurred predominantly at L4–L5 and L5–S1 levels, with no cases reported in upper lumbar segments, likely reflecting their lower utilization.

It was not possible to stratify clinical outcomes according to the presence or absence of pseudarthrosis, as only two studies [11, 15] reported VAS and/or ODI scores separately for these subgroups. Therefore, clinical improvement was analyzed for the overall ALIF cohort, based on mean differences between preoperative and postoperative values.

**Fig. 5:**
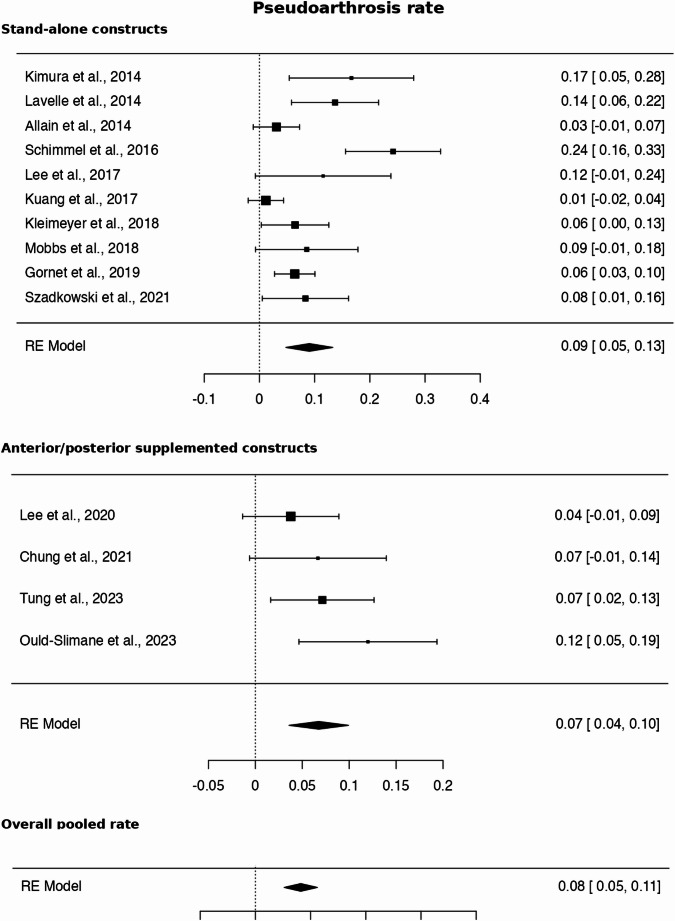
Pseudoarthrosis rate according to construct type: stand-alone vs. ALIF with posterior supplemental fixation

**Fig. 6:**
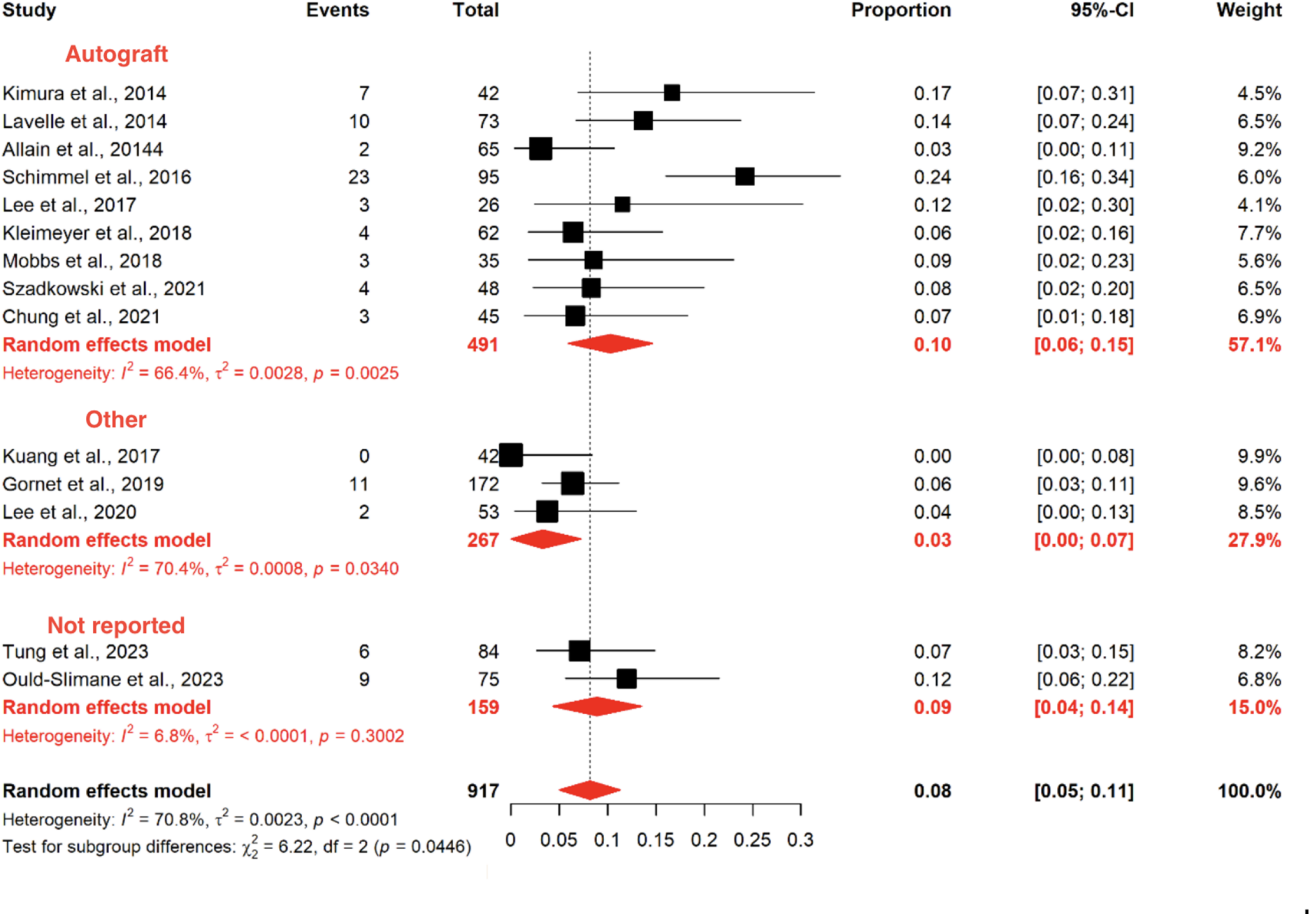
Forest plot of the meta-analysis evaluating the pooled prevalence of pseudarthrosis stratified by graft type

**Fig. 7:**
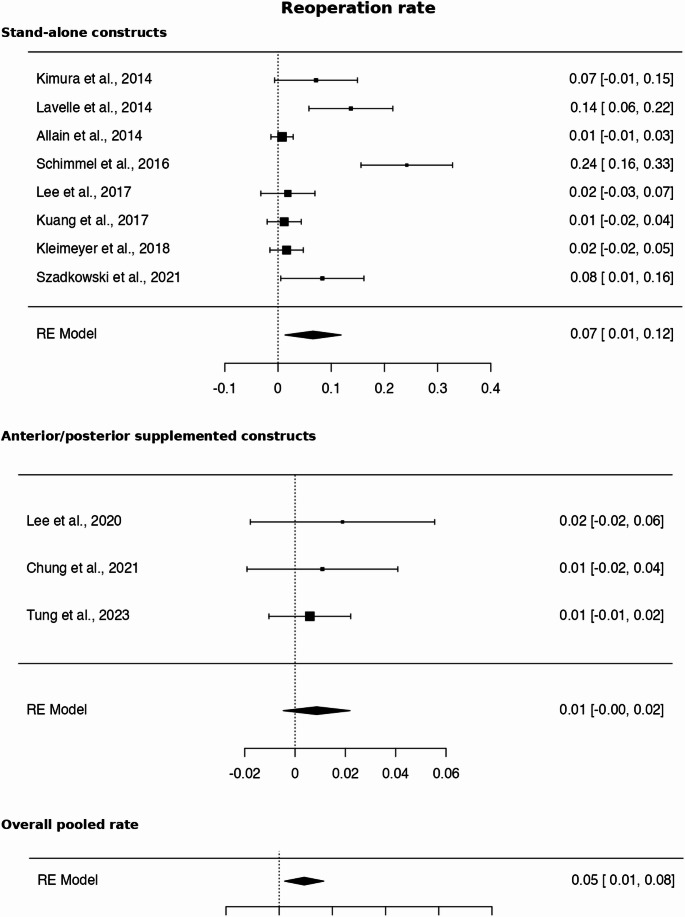
Reoperation RR of 6.8 ( 95% CI 1.9–24.5, *p* < 0.01) comparing stand-alone vs. ALIF with posterior supplemental fixation

The pooled prevalence of pseudarthrosis was 8.17% (95% CI: 5.04–11.31), with substantial heterogeneity (I² = 70.8%). Although rates were numerically higher in stand-alone ALIF compared to anterior-posterior constructs, this difference was not statistically significant (Table 5).

**Table 5:** Mantel–Haenszel pooled analysis (fixed-effect) demonstrated a relative risk of pseudoarthrosis of 1.28 (95% CI 0.78–2.08; *p* = 0.32) comparing stand-alone vs. ALIF with posterior supplemental fixation

	Pseudoarthrosis (Yes)	Pseudoarthrosis (No)	Total
Stand-alone = Yes	67	593	660
Stand-alone = No posterior supplemental fixation	20	237	*257*
Total	*87*	*830*	***917***

Similarly, no significant differences were observed according to the number of fused levels. In contrast, subgroup analysis by graft type demonstrated a statistically significant difference, with higher pseudarthrosis rates in studies using autologous grafts compared to alternative grafts (*p* = 0.0446). However, this finding should be interpreted with caution, as a substantial proportion of the alternative graft group included the use of bone morphogenetic proteins (BMPs), which are known to enhance fusion rates and may have influenced these results.

Detailed results are presented in Table 6, Figs. 5 and 6.

**Table 6:** Pooled prevalence of pseudarthrosis across included studies and subgroup analyses

Label	Studies (*n*)	Cases (*n*)	Pseudarthrosis (*n*)	Pooled Prevalence (95% CI)	Heterogeneity (I²)	*p*-value
Overall model						
Kimura et al., 2014 [11]	1	42	7	16.67 (6.97–31.36)	–	–
Lavelle et al., 2014 [12]	1	73	10	13.70 (6.77–23.75)	–	–
Allain et al., 2014 [13]	1	65	2	3.08 (0.37–10.68)	–	–
Schimmel et al., 2016 [15]	1	95	23	24.21 (16.01–34.08)	–	–
Lee et al., 2017 [16]	1	26	3	11.54 (2.45–30.15)	–	–
Kuang et al., 2017 [17]	1	42	0	0.00 (0–8.41)	–	–
Kleimeyer et al., 2018 [18]	1	62	4	6.45 (1.79–15.70)	–	–
Mobbs et al., 2018 [19]	1	35	3	8.57 (1.80–23.06)	–	–
Gornet et al., 2019 [20]	1	172	11	6.40 (3.24–11.15)	–	–
Szadkowski et al., 2021 [21]	1	48	4	8.33 (2.32–19.98)	–	–
Lee et al., 2020 [14]	1	53	2	3.77 (0.46–12.98)	–	–
Chung et al., 2021 [22]	1	45	3	6.67 (1.40–18.27)	–	–
Tung et al., 2023 [23]	1	84	6	7.14 (2.67–14.90)	–	–
Ould-Slimane et al., 2023 [24]	1	75	9	12.00 (5.64–21.56)	–	–
Pooled estimate	14	917	87	8.17 (5.04–11.31)	70.8%	< 0.001
Subgroup Analysis						
Label	**Studies (n)**	**Cases (n)**	**Pseudarthrosis (n)**	**Pooled Prevalence (95% CI)**	**I²**	**p-value**
Fixation type						
Stand-alone (no screws)	10	660	67	8.95 (4.54–13.37)	78.0%	< 0.001
Anterior-posterior (with pedicle screws)	4	257	20	6.76 (3.58–9.94)	8.3%	352
Test for subgroup differences	–	–	–	–	–	429
Label	**Studies (n)**	**Cases (n)**	**Pseudarthrosis (n)**	**Pooled Prevalence (95% CI)**	**I²**	**p-value**
Number of fused levels						
Single level	7	473	39	7.45 (4.42–10.49)	30.6%	< 0.001
One or two levels	6	391	46	9.45 (2.91–15.98)	85.5%	< 0.001
Two levels	1	53	2	3.77 (0–8.90)	–	–
Test for subgroup differences	–	–	–	–	–	343
Label	**Studies (n)**	**Cases (n)**	**Pseudarthrosis (n)**	**Pooled Prevalence (95% CI)**	**I²**	**p-value**
Number of levels (grouped)						
Single level	7	473	39	7.45 (4.42–10.49)	30.6%	< 0.001
One or more levels	7	444	48	8.50 (2.86–14.14)	82.7%	< 0.001
Test for subgroup differences	–	–	–	–	–	749
Label	**Studies (n)**	**Cases (n)**	**Pseudarthrosis (n)**	**Pooled Prevalence (95% CI)**	**I²**	**p-value**
Graft type						
Autograft	9	491	59	10.31 (5.94–14.69)	66.4%	2
Bioceramic	1	42	0	0.00 (0–3.20)	–	–
BMP	1	172	11	6.40 (2.74–10.05)	–	–
Allograft	1	53	2	3.77 (0–8.90)	–	–
Not reported	2	159	15	8.93 (4.34–13.53)	6.8%	300
Test for subgroup differences	–	–	–	–	–	1
Label	**Studies (n)**	**Cases (n)**	**Pseudarthrosis (n)**	**Pooled Prevalence (95% CI)**	**I²**	**p-value**
Graft type (grouped)						
Autograft	9	491	59	10.31 (5.94–14.69)	66.4%	2
Other (BMP, bioceramic, allograft)	3	267	13	3.27 (0–7.26)	70.4%	34
Not reported	2	159	15	8.93 (4.34–13.53)	6.8%	300
Test for subgroup differences	–	–	–	–	–	446

### Reoperation analysis

Pseudoarthrosis rates did not demonstrate a statistically significant difference between groups. However, when analyzing clinically relevant failure—defined as reoperation for symptomatic pseudoarthrosis—patients who underwent ALIF with posterior supplemental fixation exhibited significantly lower reoperation rates. In contrast, stand-alone ALIF was associated with a markedly increased risk of reoperation, with a relative risk of 6.8 (*p* < 0.01) (Table 7; Fig. 7).

**Table 7:** Mantel–Haenszel pooled analysis demonstrated a relative risk of RR of 6.8 ( 95% CI 1.9–24.5, *p* < 0.01) comparing stand-alone vs. ALIF with posterior supplemental fixation

Stand-alone = Yes	Reoperation (Yes)	Reoperation (No)	Total
	41	412	453
Stand-alone = No posterior supplemental fixation	1	181	*182*
Total	*42*	*593*	***635***

### Other complications

Regarding additional complications, the included studies reported a total of 41 cases of cage subsidence, 34 access-related complications (including vascular injury, dysautonomia, retrograde ejaculation, deep abscess, and incisional hernia), and 32 cases of adjacent segment degeneration.

Furthermore, 24 systemic complications were reported, including surgical site infection, paralytic ileus, hematoma, and urinary retention. Less frequent events included 6 cases of cage migration, 4 cases of deformity, and 2 cases of osteoporosis.

Importantly, no deaths were attributed to the procedure or its associated complications.

## Discussion

The diagnosis of pseudarthrosis varied across studies, with computed tomography (CT) being the most commonly used and likely the most reliable modality, given its superior sensitivity and specificity compared to radiographs. Radiological findings such as loss of disc height, implant failure, graft resorption, and peri-implant radiolucency were consistently reported as indicators of nonunion.

The overall pseudarthrosis rate (8.17%) was consistent with previously reported data [25–27], and no statistically significant differences were observed between stand-alone ALIF and anterior-posterior constructs, suggesting comparable fusion performance between techniques. However, these findings should be interpreted with caution, as surgical decision-making is strongly influenced by baseline instability; in clinical practice, patients with degenerative spondylolisthesis, segmental instability, deformity, or multilevel disease are more likely to undergo ALIF with posterior supplemental fixation [28], introducing potential indication bias. Importantly, despite similar radiographic pseudarthrosis rates, clinically meaningful differences emerged when reoperation was considered, with stand-alone ALIF associated with a significantly higher risk of reoperation for symptomatic pseudarthrosis, whereas circumferential constructs demonstrated greater mechanical reliability [28]. Furthermore, no significant association was found between pseudarthrosis rates and improvements in VAS or ODI, reinforcing that radiographic nonunion does not necessarily correlate with worse clinical outcomes. This supports the concept that pseudarthrosis is a heterogeneous condition, in which only a small subset of patients—those with so-called “clinical pseudarthrosis”—develop symptoms requiring intervention.

From a clinical perspective, both techniques were associated with significant improvements in pain and functional outcomes, with no differences according to fixation strategy, number of fused levels, or graft type. Regarding graft selection, autologous bone graft remains the gold standard due to its osteogenic, osteoinductive, and osteoconductive properties [29, 30], and the higher pseudarthrosis rates observed likely reflect confounding factors, as well as the use of alternative grafts such as bone morphogenetic protein (BMP) [29–32]. In addition, implant characteristics, including cage material, may influence biomechanical behavior and fusion environment, with studies comparing polyetheretherketone (PEEK) and titanium cages demonstrating differences in subsidence and fusion-related outcomes [31, 33, 34].

 Most procedures involved single-level fusion at L4–L5 and L5–S1, consistent with standard indications, whereas multilevel procedures were more frequently supplemented with posterior fixation due to increased instability [35]. Patient-related factors, including demographic characteristics and sex-specific differences in degenerative lumbar disease, may also influence surgical indication and outcomes [36, 37].

Overall, these findings suggest that stand-alone ALIF and circumferential fusion provide similar radiographic and clinical outcomes in appropriately selected patients; however, supplemental posterior fixation may play a critical role in a specific subgroup at higher risk of clinically relevant nonunion. Future studies should focus on identifying these patients—particularly those with greater biomechanical instability, smoking history, or compromised bone quality—to define better which cases may benefit from routine posterior supplementation and to support a more individualized, risk-based surgical strategy.

### Limitations

This study has several important limitations. First, the analysis was predominantly based on aggregated data, precluding patient-level comparisons between pseudarthrosis and solid fusion in terms of pain (VAS), functional outcomes (ODI), and quality of life.

An additional methodological limitation concerns the lack of standardized criteria for defining fusion success across included studies. While the majority utilized CT-based assessment of bony bridging, definitions varied among studies and no universal radiographic threshold was applied. Furthermore, none of the included studies performed quantitative measurement of interspinous motion to determine fusion status; dynamic radiographs were used qualitatively when reported. Time-to-pseudoarthrosis data were unavailable in all studies, as pseudoarthrosis was reported as a binary outcome at final follow-up rather than as a time-dependent event. Consequently, stratified analysis by time to pseudoarthrosis could not be performed. These factors limit the precision of our pooled estimates and should be considered when interpreting the comparative outcomes between stand-alone and supplemented ALIF.

The available evidence is largely derived from retrospective cohort studies, with a notable scarcity of randomized controlled trials, limiting the strength of causal inferences. In addition, there was substantial clinical heterogeneity across studies, including differences in patient populations (age, underlying pathology), surgical indications, number of treated levels, and graft materials (autograft, BMP, allograft, and substitutes). This variability likely contributed to the high heterogeneity observed in the analyses and limits the comparability of pooled results.

Subgroup analyses, particularly those comparing stand-alone constructs versus posteriorly supplemented ALIF, were further limited by imbalances in study numbers and potential indication bias, as more complex or unstable cases are more likely to receive supplemental fixation. Finally, inconsistencies in outcome definitions and measurement methods, along with limited control of confounding factors, may have influenced the results and should be considered when interpreting the findings.

## Conclusion

Anterior lumbar interbody fusion (ALIF) for degenerative lumbar disc disease provides significant improvements in pain and functional outcomes, even in the presence of pseudarthrosis. Although stand-alone ALIF and circumferential fusion demonstrated comparable fusion rates and overall clinical results, the addition of posterior pedicle screw fixation was associated with lower reoperation rates related to symptomatic nonunion, suggesting a more durable and mechanically reliable construct.

These findings support the use of both techniques as clinically effective strategies; however, supplemental posterior fixation may be particularly advantageous in selected patients at higher risk of fusion failure. Future studies focused on patient stratification and precision-based approaches are needed to better define which clinical and biomechanical profiles are most susceptible to symptomatic pseudarthrosis, thereby guiding individualized surgical decision-making.

## Electronic Supplementary Material

Below is the link to the electronic supplementary material.

Appendix 1 (DOCX 7.81 KB)

Appendix 2 (DOCX 81.3 KB)

## Data Availability

All data analyzed in this study are derived from previously published articles, which are cited in the manuscript. The datasets generated and/or analyzed during the current study are available from the corresponding author on reasonable request.

## Abbreviations

ALIF: Anterior Lumbar Interbody Fusion
PLIF: Posterior Lumbar Interbody Fusion
TLIF: Transforaminal Lumbar Interbody Fusion
LLIF: Lateral Lumbar Interbody Fusion
OLIF: Oblique Lumbar Interbody Fusion
ODI: Oswestry Disability Index
VAS: Visual Analog Scale
BMP: Bone Morphogenetic Protein
CT: Computed Tomography
MRI: Magnetic Resonance Imaging
PRISMA: Preferred Reporting Items for Systematic Reviews and Meta–Analyses
PROSPERO: International Prospective Register of Systematic Reviews
PICO: Population, Intervention, Comparison, Outcome
JBI: Joanna Briggs Institute
RoB 2: Risk of Bias 2 tool
CI: Confidence Interval
RR: Relative Risk
SD: Standard Deviation
RCT: Randomized Controlled Trial
