# Supplementary material for: Stand-alone versus supplemented ALIF: a systematic review and meta-analysis of pseudarthrosis and reoperation rates
Source: Neurosurg Rev. 2026 Jun 3;49(1):430. doi: 10.1007/s10143-026-04347-1 (PMC13233657; doi:10.1007/s10143-026-04347-1)
Supplement: Supplementary file 1 — Appendix 1 (DOCX 7.81 KB) [file 10143_2026_4347_MOESM1_ESM.docx]

**Appendix 1. Search Strategy**

The literature search was performed on September 24, 2024, in PubMed/MEDLINE, Embase, Cochrane Library, and BVS. The search strategy was designed to identify studies evaluating anterior lumbar interbody fusion (ALIF), particularly stand-alone ALIF and ALIF supplemented with posterior pedicle screw fixation, with emphasis on pseudarthrosis, fusion failure, and related radiological outcomes. Controlled vocabulary terms and free-text keywords were combined using Boolean operators. No language restrictions were applied during the initial search. The search was restricted to studies published from January 2013 to September 2024.

**PubMed/MEDLINE**

Search date: September 24, 2024
Time frame: January 2013 to September 2024
Records identified: 185

Search strategy:

(("Anterior Lumbar Interbody Fusion"[Mesh] OR "anterior lumbar interbody fusion"[Title/Abstract] OR "ALIF"[Title/Abstract] OR "lumbar interbody fusion"[Title/Abstract] OR "anterior interbody fusion"[Title/Abstract])
AND
("Pseudarthrosis"[Mesh] OR pseudarthrosis[Title/Abstract] OR pseudoarthrosis[Title/Abstract] OR "nonunion"[Title/Abstract] OR "non-union"[Title/Abstract] OR "fusion failure"[Title/Abstract] OR "failed fusion"[Title/Abstract] OR "lack of fusion"[Title/Abstract] OR "arthrodesis failure"[Title/Abstract])
AND
("Lumbar Vertebrae"[Mesh] OR lumbar[Title/Abstract] OR lumbosacral[Title/Abstract])
AND
("Degenerative Disc Disease"[Title/Abstract] OR degenerative[Title/Abstract] OR "spondylolisthesis"[Mesh] OR spondylolisthesis[Title/Abstract] OR "spinal stenosis"[Mesh] OR stenosis[Title/Abstract] OR "disc degeneration"[Title/Abstract]))

Filters applied: publication date from 2013/01/01 to 2024/09/24.

**Embase**

Search date: September 24, 2024
Time frame: January 2013 to September 2024
Records identified: 317

Search strategy:

('anterior lumbar interbody fusion'/exp OR 'anterior lumbar interbody fusion',ab OR alif,ab OR 'lumbar interbody fusion',ab OR 'anterior interbody fusion',ab)
AND
('pseudarthrosis'/exp OR pseudarthrosis,ab OR pseudoarthrosis,ab OR nonunion,ab OR 'non union',ab OR 'non-union',ab OR 'fusion failure',ab OR 'failed fusion',ab OR 'lack of fusion',ab OR 'arthrodesis failure',ab)
AND
('lumbar spine'/exp OR lumbar,ab OR lumbosacral,ab)
AND
('degenerative disc disease'/exp OR degenerative,ab OR 'spondylolisthesis'/exp OR spondylolisthesis,ab OR 'spinal stenosis'/exp OR stenosis,ab OR 'disc degeneration',ab)

Limits applied: 2013 to 2024.

**Cochrane Library**

Search date: September 24, 2024
Time frame: January 2013 to September 2024
Records identified: 71

Search strategy:

("anterior lumbar interbody fusion" OR ALIF OR "lumbar interbody fusion" OR "anterior interbody fusion")
AND
(pseudarthrosis OR pseudoarthrosis OR nonunion OR "non-union" OR "fusion failure" OR "failed fusion" OR "lack of fusion" OR "arthrodesis failure")
AND
(lumbar OR lumbosacral)
AND
(degenerative OR "degenerative disc disease" OR spondylolisthesis OR stenosis OR "spinal stenosis" OR "disc degeneration")

Limits applied: publication date from January 2013 to September 2024.

**BVS / Biblioteca Virtual em Saúde**

Search date: September 24, 2024
Time frame: January 2013 to September 2024
Records identified: 212

Search strategy:

(("anterior lumbar interbody fusion" OR ALIF OR "lumbar interbody fusion" OR "anterior interbody fusion" OR "fusão intersomática lombar anterior" OR "fusão lombar anterior")
AND
(pseudarthrosis OR pseudoarthrosis OR nonunion OR "non-union" OR "fusion failure" OR "failed fusion" OR "arthrodesis failure" OR pseudoartrose OR pseudartrose OR "falha de fusão" OR "falha da artrodese")
AND
(lumbar OR lumbosacral OR lombar OR lombossacral)
AND
(degenerative OR "degenerative disc disease" OR spondylolisthesis OR stenosis OR "spinal stenosis" OR "disc degeneration" OR degenerativa OR "doença degenerativa do disco" OR espondilolistese OR estenose))

Limits applied: publication date from January 2013 to September 2024.

Across all databases, the references retrieved were exported to a reference manager, and duplicate records were removed before title and abstract screening. The final search yielded 785 records before duplicate removal: PubMed/MEDLINE (n = 185), Embase (n = 317), Cochrane Library (n = 71), and BVS (n = 212).
