# Supplementary material for: Stand-alone versus supplemented ALIF: a systematic review and meta-analysis of pseudarthrosis and reoperation rates
Source: Neurosurg Rev. 2026 Jun 3;49(1):430. doi: 10.1007/s10143-026-04347-1 (PMC13233657; doi:10.1007/s10143-026-04347-1)
Supplement: Supplementary file 2 — Appendix 2 (DOCX 81.3 KB) [file 10143_2026_4347_MOESM2_ESM.docx]

**Appendix 2. Risk of Bias Assessment**

The methodological quality and risk of bias of the included studies were independently assessed by two reviewers according to study design.

For randomized controlled trials (RCTs), the Cochrane Risk of Bias 2 (RoB 2) tool was used to evaluate potential bias across the following domains: randomization process, deviations from intended interventions, missing outcome data, outcome measurement, and selection of the reported results. The overall assessment is summarized in Figure 2.

Both included RCTs were judged to present a high overall risk of bias. The principal concerns were related to incomplete or insufficiently described randomization methods, missing outcome data with potential impact on the validity of results, and limited transparency regarding selective reporting of outcomes. In addition, the inherent challenges of blinding in surgical intervention studies may have contributed to performance and detection bias.

The methodological quality of observational studies was assessed using the Joanna Briggs Institute (JBI) Critical Appraisal Checklist for Cohort Studies. Each study was evaluated regarding participant selection, measurement of exposure and outcomes, identification and management of confounding factors, adequacy of follow-up, and appropriateness of statistical analysis. The detailed assessment is presented in Table 2.

Overall, the observational studies demonstrated moderate to high methodological quality, with most appraisal domains adequately addressed. However, important limitations were identified, particularly regarding incomplete adjustment for confounding variables, heterogeneity in patient selection criteria, variability in definitions of fusion and pseudarthrosis, and differences in radiological assessment methods and follow-up duration. These factors may have introduced residual confounding and contributed to methodological heterogeneity across studies.

Despite these limitations, the included studies were considered sufficiently robust to allow qualitative synthesis and quantitative meta-analytic evaluation.


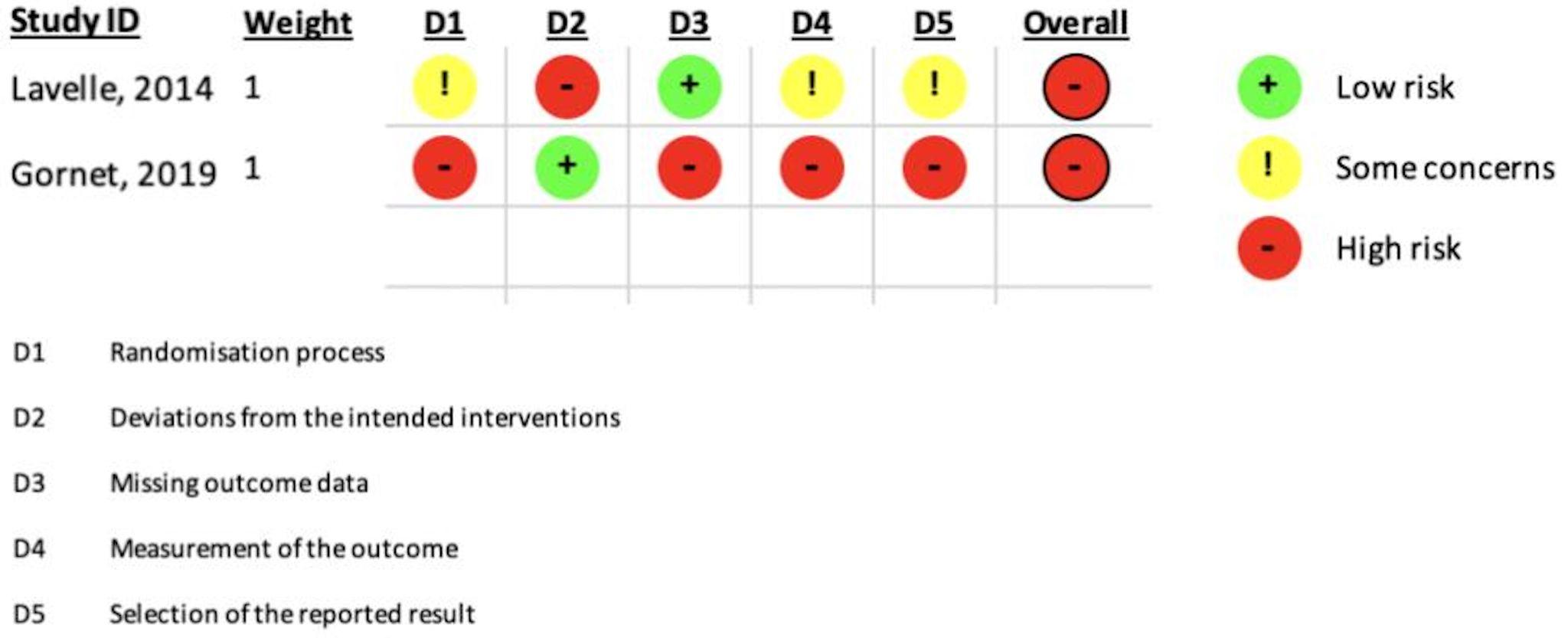


**Figure 2.** ROB2 assessment of the RCTs.

**Table 2.** Risk of bias assessment of included studies using the Joanna Briggs Institute (JBI) Critical Appraisal Tool for observational studies.

|  | **Risk of Bias Assessment - JBI** | | | | | | | | | | | | | **Yes** | **No** | **Not clear** |
| --- | --- | --- | --- | --- | --- | --- | --- | --- | --- | --- | --- | --- | --- | --- | --- | --- |
| **ID** | **1** | **2** | **3** | **4** | **5** | **6** | **7** | **8** | **9** | **10** | **11** | **12** | **13** |  |  |  |
| Kimura et al., 2014 | S | S | S | NC | NC | S | S | S | No | S | - | - | - | 70,0% | 10,0% | 20,0% |
| Allain et al., 20144 | S | S | S | NC | NC | S | NC | NC | No | S | - | - | - | 50,0% | 10,0% | 40,0% |
| Lee et al., 2020 | S | S | S | NC | NC | S | S | S | S | NC | S | - | - | 72,7% | 0,0% | 27,3% |
| Schimmel et al., 2016 | S | S | S | NC | NC | S | S | S | No | S | - | - | - | 70,0% | 10,0% | 20,0% |
| Lee et al., 2017 | S | S | S | No | No | S | S | S | S | NC | S | - | - | 72,7% | 18,2% | 9,1% |
| Kuang et al., 2017 | S | S | S | No | NC | S | S | S | S | - | S | - | - | 80,0% | 10,0% | 10,0% |
| Kleimeyer et al., 2018 | S | S | S | NC | NC | S | S | S | S | No | S | - | - | 72,7% | 9,1% | 18,2% |
| Mobbs et al., 2018 | S | S | S | S | S | No | S | S | No | S | - | - | - | 80,0% | 20,0% | 0,0% |
| Szadkowski et al., 2021 | NC | S | S | S | S | S | S | S | NC | S | - | - | - | 80,0% | 0,0% | 20,0% |
| Chung et al., 2021 | S | S | S | No | No | S | S | S | S | - | S | - | - | 80,0% | 20,0% | 0,0% |
| Tung et al., 2023 | S | S | S | S | NC | S | S | S | NC | S | - | - | - | 80,0% | 0,0% | 20,0% |
| Ould-Slimane et al., 2023 | S | S | S | NC | NC | S | S | S | No | S | - | - | - | 70,0% | 10,0% | 20,0% |
| **Yes** | 92,9% | 85,7% | 100% | 21,4% | 14,3% | 92,9% | 78,6% | 92,9% | 50,0% | 66,7% | 100% | 100% | 100% |  |  |  |
| **No** | 0,0% | 0,0% | 0,0% | 35,7% | 28,6% | 7,1% | 14,3% | 0,0% | 35,7% | 16,7% | 0,0% | 0,0% | 0,0% |  |  |  |
| **Not clear** | 7,1% | 14,3% | 0,0% | 42,9% | 57,1% | 0,0% | 7,1% | 7,1% | 14,3% | 16,7% | 0,0% | 0,0% | 0,0% |  |  |  |
